# Steel Slag Accelerated Carbonation Curing for High-Carbonation Precast Concrete Development

**DOI:** 10.3390/ma17122968

**Published:** 2024-06-17

**Authors:** Weilong Li, Hui Wang, Zhichao Liu, Ning Li, Shaowei Zhao, Shuguang Hu

**Affiliations:** 1 State Key Laboratory of Silicate Materials for Architectures, Wuhan University of Technology, Wuhan 430070, China; weilongli@whut.edu.cn (W.L.); hsglab@whut.edu.cn (S.H.); 2 State Key Laboratory of Solid Waste Reuse for Building Materials, Beijing Building Materials Academy of Science Research, Beijing 100041, China; wanghui@bbma.com.cn (H.W.); liqingning123456@126.com (N.L.); zhaoshaowei5138@163.com (S.Z.)

**Keywords:** steel slag, carbonation, durability, freeze–thaw cycle, high-temperature resistance

## Abstract

Steel slag as an alkaline industrial solid waste, possesses the inherent capacity to engage in carbonation reactions with carbon dioxide (CO_2_). Capitalizing on this property, the current research undertakes a systematic investigation into the fabrication of high-carbonation precast concrete (HCPC). This is achieved by substituting a portion of the cementitious materials with steel slag during the carbonation curing process. The study examines the influence of varying water–binder ratios, silica fume dosages, steel slag dosages, and sand content on the compressive strength of HCPC. Findings indicate that adjusting the water–binder ratio to 0.18, adding 8% silica fume, and a sand volume ratio of 40% can significantly enhance the compressive strength of HCPC, which can reach up to 104.9 MPa. Additionally, the robust frost resistance of HCPC is substantiated by appearance damage analysis, mass loss rate, and compressive strength loss rate, after 50 freeze–thaw cycles the mass loss, and the compressive strength loss rate can meet the specification requirements. The study also corroborates the high-temperature stability of HCPC. This study optimized the preparation of HCPC and provided a feasibility for its application in precast concrete.

## 1. Introduction

The swift expansion of the steel industry has positioned China as the preeminent global producer of steel, commanding a market share exceeding 50% of the world’s crude steel production [1,2]. In the steelmaking process, a substantial 10–15% of the total volume is converted into steel slag [3], a byproduct of the iron and steel industry that significantly contributes to the metallurgical sector’s solid waste output [4,5]. As of 2023, China’s steel production has soared to 1.363 billion tons, with crude steel accounting for 1.019 billion tons, and a figure that continues to escalate annually. However, limitations in manufacturing equipment, preparation methods, and geographic location have constrained the physical properties and stability of steel slag, leading to its low utilization rate of less than 30% [6,7]. As a consequence, the majority of this byproduct is relegated to open-air stockpiles, presenting considerable environmental hazards and encroaching upon arable land [8].

As global industrialization accelerates, the repercussions of climate change are becoming increasingly pronounced. In the context of China’s advancement towards an ecological civilization, the notions of carbon emissions and carbon neutrality have attracted considerable interest. In a modest commitment, the Chinese government set forth the “Dual Carbon” Goal in 2020, aiming for a carbon peak by 2030 and achieving carbon neutrality by 2060 [9,10,11]. Among the array of potential solutions, Carbon Capture, Utilization, and Storage (CCUS) emerges as a pivotal technology, capable of markedly curtailing CO_2_ emissions derived from the combustion of fossil fuels. There exists an exigent demand for the innovation of novel carbon capture technologies [12]. Concurrently, promoting the efficient use of steel slag becomes essential within the “carbon peak, carbon neutral” framework to ensure the sustainable development of Chinese society [13].

Mineral carbonation is recognized as a viable approach for the storage and sequestration of carbon [14]. This natural process is exemplified by the formation of stalactites, which involve the slow solidification of CO_2_ by natural calcium/magnesium minerals. Interestingly, the primary mineral components of steel slag resemble those found in cement, including phases like C_3_S, C_2_S, and C_4_AF. Additionally, steel slag contains minor amounts of C_2_F and various oxides such as magnesium, iron, and manganese, which are products of the solid solution of the RO-phase and other non-cementitious minerals [15]. The high carbonation reactivity of steel slag is attributed to its content of calcium hydroxide and silicates [6,15]. The rapid carbonation process of steel slag precipitates chemically stable carbonates, which results in a swift enhancement of its mechanical strength. Moreover, the application of CO_2_ treatment can neutralize free lime (f-CaO) and magnesia (f-MgO) present in the slag, thereby augmenting its overall stability [16].

Prefabrication is an initiative that has been established to improve the efficiency and sustainability of the building industry [17]. A strategy to building called prefabrication involves assembling building components on construction sites after they have been manufactured in specialized facilities [18]. Prefabrication provides the advantages of high construction efficiency, low prices, high product quality, and higher environmental performance when compared to conventional construction methods [19,20]. Studies have categorized the carbonation curing of steel slag into two main methods based on the molding technique: compression molding and pouring molding [14]. The hardened structure and the performance post-carbonation curing exhibit significant variances between the two molding techniques. The process of compression molding for steel slag obviates the need for powder fluidity, enabling the fabrication of a compact billet through direct compaction under controlled pressure and moisture conditions [8,21,22,23,24]. Despite its ability to produce dense materials, this method is constrained to uniform product dimensions and is associated with considerable production costs due to its stringent process requirements. Conversely, pouring molding samples commonly utilize higher water-to-solid ratios, ranging from 0.3 to 0.6, to ensure adequate mix fluidity [16,25,26,27,28]. However, an overabundance of moisture at this stage can impede the penetration of CO_2_ during the curing process, potentially resulting in diminished compressive strength following carbonation. Nonetheless, pouring molding offers distinct advantages for the production of carbonized steel slag products, such as the flexibility to manufacture a diverse array of sizes and shapes through the use of templates, along with a more streamlined preparation process.

This study introduces high-carbonation precast concrete (HCPC), engineered by optimizing the particle size distribution of raw materials to create a denser matrix, drawing inspiration from the properties of ultra-high-performance concrete (UHPC). High compressive strength and good resistance to chloride ion penetration can be achieved in a relatively short period of carbonation curing by means of cast molding. It systematically examines the impact of varying water-to-binder (w/b) ratios, silica fume dosages, steel slag contents, and sand proportions on the compressive strength of HCPC, revealing the underlying patterns of strength development. The research then shifts focus to the durability aspects of HCPC, assessing its resistance to chloride attack, the effects of freeze–thaw cycles on mass and strength loss, and its thermal stability. The results shed new light on the potential of steel slag as an effective carbonation binder and for use in industrial precast concrete.

## 2. Experimental

### 2.1. Raw Materials and Characterization

**Figure 1:**
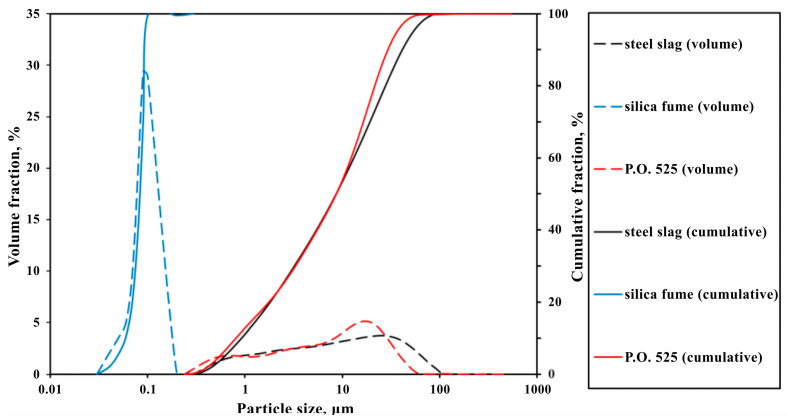
Particle size distribution of cement, steel slag, and silica fume.

**Figure 2:**
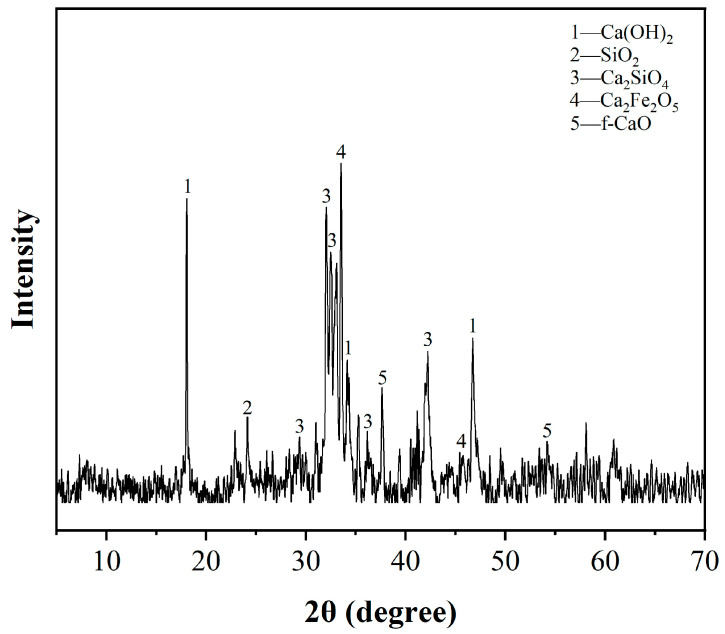
XRD spectrum of CSS.

In this study, the converted steel slag (CSS) was sourced from the China Baowu Steel Group in Shanghai, China. Ordinary silicate cement of P.O 52.5, provided by Huaxin Cement Corporation, Wuhan, China, and silica fume (SF) from Chengdu East Blue-star Science and Technology Development Corporation, Chengdu, China, were also used. Figure 1 illustrates the particle size distribution of these materials, revealing that while cement and CSS have similar distributions, SF has the smallest particles. The XRD spectrum in Figure 2 indicates that CSS primarily consists of C_2_S, C_3_S, C_2_F, and the RO phase—a solid solution of oxides like magnesium, iron, and manganese—along with minor amounts of Ca(OH)_2_, f-CaO, and SiO_2_. Using the glycerol ethanol method, the f-CaO content in the steel slag was determined to be 9.3 wt.%. Table 1 presents the chemical compositions of the cement, CSS, and SF. Quartz sand with a fineness modulus of 2.74, as measured by sieving, was used as the aggregate. The water-reducing agent (SP) employed was a polycarboxylic acid-based high-efficiency reducer with a 38% solid content, supplied by Huaxin Corporation, Wuhan, China.

### 2.2. Experimental

#### 2.2.1. Mixing Ratio Design

The mixing ratio for high-carbonation performance concrete (HCPC) was adapted from that of ultra-high-performance concrete (UHPC), focusing on the tight packing of raw materials. However, Table 2 shows that a portion of the cement was replaced by converted steel slag (CSS) on a mass basis. The mix designs varied the water-to-binder (w/b) ratio, SF dosages, CSS content, and sand proportion using a controlled variable approach. To achieve the desired workability, the SP water reducer was used to extend the fluidity of HCPC to over 190 ± 10 mm. In these mixtures, CSS served as the primary binder, with supplemental cement and SF (up to 20 wt.%) incorporated to leverage the high hydration reactivity of the cement for early strength development and the reinforcing properties of SF to enhance matrix densification.

#### 2.2.2. Specimen Preparation

**Figure 3:**
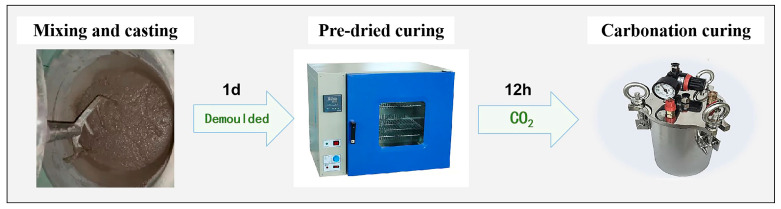
Specimen preparation process.

HCPC was prepared according to the mixing ratio, then cast and molded into 40 × 40 × 40 mm cubic specimens, which were used for compressive strength testing, rectangular specimens of 40 × 40 × 160 mm were used for freeze–thaw cycles, and cylinders of φ100 × 50 mm were used for resistance to chloride erosion. These were sealed in their molds and cured in a constant temperature room at 20 ± 2 °C for one day. After demolding, the specimens were pre-dried in a blast drying oven at 40 °C for 12 h before being placed in a carbonation pressure reactor for further curing. The curing process began by opening the reactor’s outlet valve and flushing the reactor with a continuous flow of 99.9% CO_2_ for 30 s to expel any air. The outlet valve was then closed, and CO_2_ injection continued until the internal pressure reached 0.4 MPa, exceeding the external atmospheric pressure. After 12 h of carbonation curing, the specimens were removed for testing. Figure 3 provides a schematic representation of the specimen preparation process.

### 2.3. Testing Methods

A Mastersizer 3000 ultra-high-speed intelligent particle size analyzer was used for the particle size test, manufactured by Malvern, Malvern, UK. The particle size analyzer had a measurement range of 0.1 μm to 3500 μm. Prior to every test, the material was ultrasonically dispersed for two minutes. For every sample that was examined, three tests were run, and the test result was calculated as the average of the three tests. A TYE-300F compressive testing device was used with a loading rate of 2.0 kN/s. Three test blocks were tested for each age group, and the test results were used to obtain the average value. A D8 Advance X-ray diffractometer from Brooks (Saarbrucken, Germany) was used for the analysis of the physical phase, with a scanning angle of 5°–70°, a step size of 0.02°, and a scanning rate of 5 (°)/min. The Rapid Chloride Permeability Coefficient Measurement (RCM) test for concrete was used to quantitatively characterize the ionic attack resistance. The dimensions of the molded specimen were 100 mm in diameter and 50 mm in height. An external electric field of 30 volts was applied to the specimens via the cathode and anode electrodes. The respective soaking solutions were a 0.3 mol/L sodium hydroxide (NaOH) solution for the upper portion and a 10% mass fraction sodium chloride (NaCl) solution for the lower portion. The duration of the test was one day. The chloride diffusion coefficient of the concrete was determined based on the depth of chloride penetration using the subsequent equation:

(1) $$D_{nssm}=\frac{0.0239\times (273+T)L}{(U-2)t}\left(x_{d}-0.0238\sqrt{\frac{\left((273+T)Lx_{d}\right)}{U-2}}\right)$$

where *D_nssm_* is the fast chloride migration coefficient of the concrete specimen (×10^−12^ m^2^/s), *T* is the average temperature of the sodium chloride solution (°C), *L* is the thickness of the specimen (50 mm), *U* is the applied voltage (*V*), x_d_ is the average depth of penetration of the tested chloride ions (mm), and *t* is the duration of the test (h).

The fast-freezing method was employed to evaluate the freezing resistance of the specimens. The KDR-V5 Concrete Fast Freeze–Thaw Tester was utilized for the freeze–thaw cycling apparatus. Each cycle, which lasts for 3 h, consists of a 1 h thawing period, allowing for the completion of 8 cycles per day. The mass and ultrasonic pulse velocity of the specimens, as well as their compressive strength, were measured after every 25 cycles to assess the frost resistance of HCPC. This resistance was quantified using two parameters: the mass loss rate (Δ*ω*) and the compressive strength loss rate (Δ*f*). The rate of mass loss, which is required to be no more than 5 percent, is calculated by the following equation:

(2) $$\Delta \omega =\frac{m_{0}-m_{n}}{m_{0}}\times 100\%$$

where Δ*ω* is the mass loss rate of the specimen after n freeze–thaw cycles, %; *m*_0_ is the mass of the specimen before freeze–thaw cycles, g; *m_n_* is the mass of the specimen after *n* freeze–thaw cycles, g.

Additionally, the high-temperature resistance of HCPC was characterized by comparing the compressive strength of specimens subjected to heat treatment at temperatures ranging from 200 °C to 700 °C for 1 h with that of the untreated control group.

## 3. Results and Discussion

### 3.1. Effect of Water–Binder Ratio on Compressive Strength of HCPC

**Figure 4:**
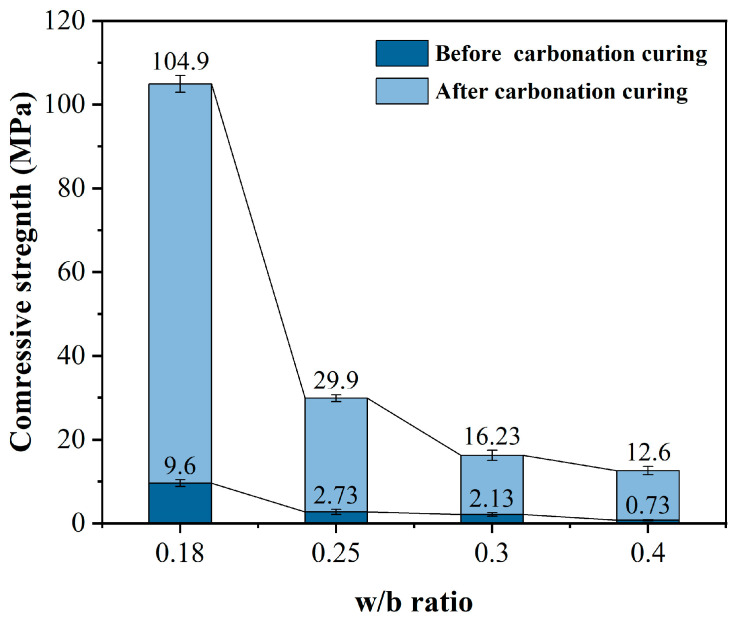
Effect of different w/b ratios on the compressive strength of HCPC before and after carbonation curing.

The influence of varying water cement ratio on the mechanical properties of HCPC was studied, both before and after carbonation. Figure 4 shows the effect of four different w/b ratios on the compressive strength of HCPC. The figure demonstrates that the compressive strength of HCPC significantly decreases as the w/b ratio increases from 0.18 to 0.4, both before and after carbonation curing. This is due to the large amount of low hydration activity CSS used, which results in excess water at higher w/b ratios. This excess water leads to increased particle spacing and a looser structure within the specimen [29]. After just one day of hydration curing, the production of hydration products is minimal, resulting in very low compressive strength before carbonation curing. The high w/b ratios also lead to a loose and porous structure, which means that the carbonation products generated after carbonation curing are insufficient to fill the internal pores of the system [30]. As a result, the compressive strength remains low even after carbonation curing. However, when the w/b ratio is reduced to 0.18, some water is consumed by cement hydration or can be evaporated during pre-dried curing. This results in a smaller internal particle spacing within the HCPC system. After further carbonation curing, the carbonation products can fill the internal pores, significantly improving the compressive strength, which can reach close to 104.9 MPa.

The appropriate reduction in the w/b ratio is shown to enhance the densification of the concrete system. A lower w/b ratio markedly augments the compressive strength post-carbonation curing. However, excessively low w/b ratios can compromise the workability of the HCPC, necessitating the incorporation of a substantial quantity of superplasticizer (SP) to regulate the fluidity [31]. Hence, a w/b ratio of 0.18 is determined to be the optimal condition for achieving a balance between mechanical performance and workability.

### 3.2. Effect of SF Dosages on Compressive Strength of HCPC

**Figure 5:**
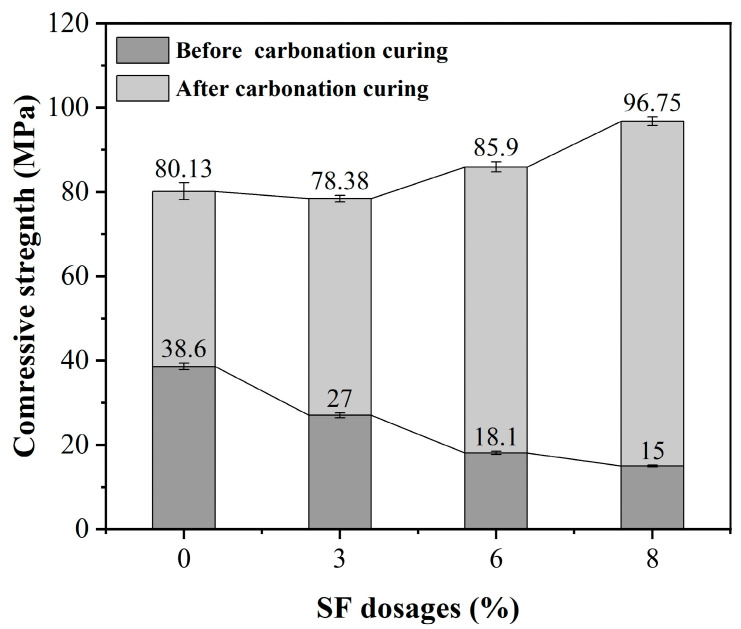
Effect of different SF dosages on the compressive strength of HCPC before and after carbonation curing.

More finer particle size of SF increases the water demand of the mixture, and an excessive amount of SF can lead to a yearly increase in the HCPC, affecting workability [32]. Shown in Figure 5 is the impact of varying SF-to-cement ratios on the compressive strength of HCPC. It is observed that as the dosage of SF increases and the cement content decreases, the compressive strength prior to carbonation curing exhibits a tendency to diminish. This indicates that while an elevated SF dosage promotes system densification, the compressive strength of the uncured specimens is predominantly dependent on cement hydration, which can result in diminished strength [32]. After carbonation curing, the compressive strength of each specimen significantly improves and continues to rise with increased SF doping. When SF levels are low and cement levels are high, early cement hydration is more pronounced, which hinders CO_2_ diffusion and migration into the system’s interior. The HCPC system’s pore structure is enhanced by a moderate inclusion of SF, and the resulting carbonation products further fill the densely packed matrix structure.

Exceeding a certain threshold in SF incorporation can adversely affect the workability of HCPC and substantially diminish its fluidity. Consequently, the incorporation of 8% SF has been identified as the optimal condition to balance the mechanical performance and workability of the concrete.

### 3.3. Effect of CSS Dosages on Compressive Strength of HCPC

**Figure 6:**
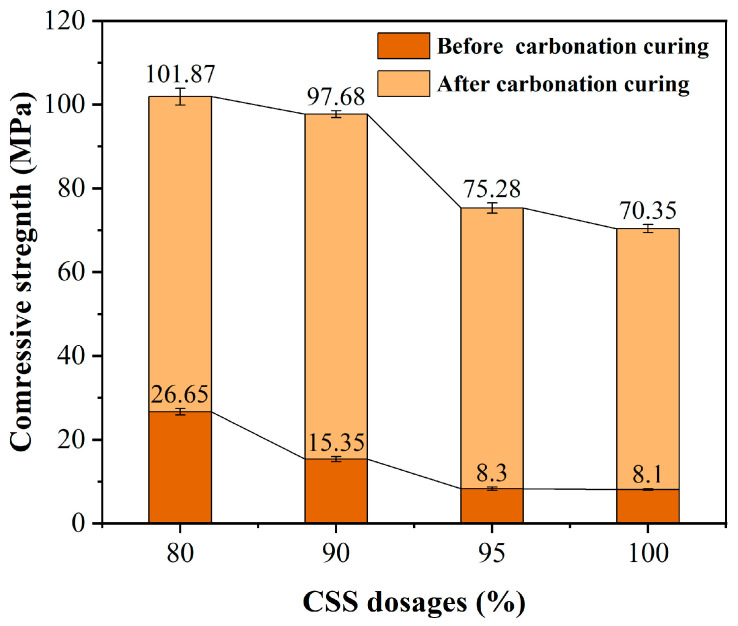
Effect of different CSS dosages on the compressive strength of HCPC before and after carbonation curing.

Figure 6 illustrates the impact of varying CSS dosages on the compressive strength of HCPC. The data reveal that an increment in CSS dosage is associated with a progressive decline in compressive strength, both before and after carbonation curing, with a more significant reduction at higher dosages. Similarly, an increased CSS dosage results in a decreased cement content within the system, leading to a reduced compressive strength prior to carbonation curing. Conversely, lower cement content also means a decreased water requirement for the system, which elevates the effective w/b ratio and enhances fluidity. Following carbonation curing, there is a discernible increase in compressive strength across all specimen groups. Nonetheless, this strength incrementally declines with escalating CSS dosages. This can be attributed to the hydration products generated by the cement-SF-CSS ternary cementitious system, which form a network skeleton structure that provides a foundation for strength development during the carbonation reaction [33]. However, when the CSS dosage is excessively high, the resulting low strength of the specimen’s hardened body before carbonation curing, coupled with insufficient strength development during curing, fails to compensate for the strength derived from early hydration.

Taking into account the maximization of CSS utilization and the optimization of concrete performance, a CSS dosage of 90% was deemed more suitable. This dosage was selected as the optimal process condition, balancing the need for high utilization of CSS with the achievement of desirable mechanical properties in the concrete.

### 3.4. Effect of Sand Content on Compressive Strength of HCPC

**Figure 7:**
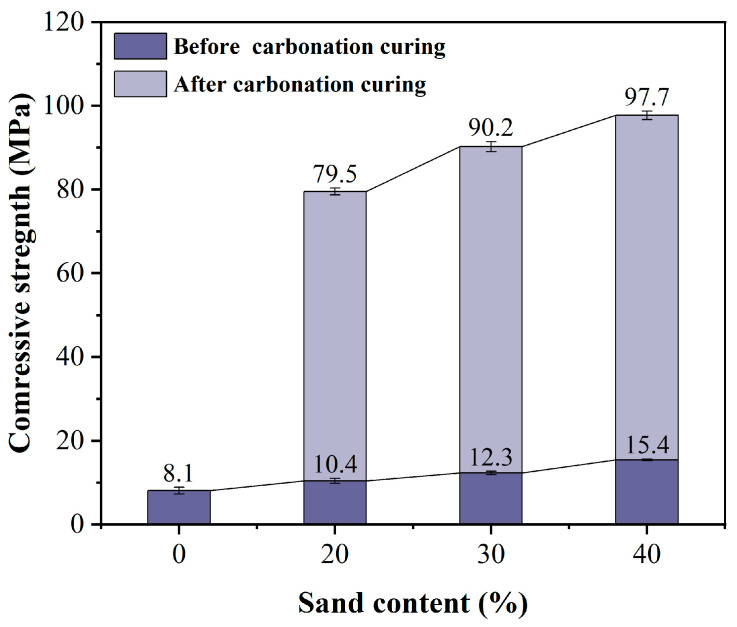
Effect of different sand content on the compressive strength of HCPC before and after carbonation curing.

Figure 7 shows the relationship between the compressive strength of HCPC and the aggregate volume fraction before and after carbonation curing. The graph clearly shows a continuous increase in compressive strength as the aggregate volume fraction rises. HCPC mixtures with 30% and 40% aggregate volume fractions have a dense system structure. The products from the carbonation reaction fill the pores, binding the hardened body into a cohesive unit, resulting in relatively high compressive strength post-curing [16]. It merits attention that specimens with a sand content of 0% result in pure slurry specimens. These pure mortar specimens undergo a vigorous exothermic reaction during carbonation curing, which induces substantial expansion and subsequent cracking. As a result, there is an absence of compressive strength data for these specimens post-curing. Moreover, the incorporation of aggregates serves to augment the overall stability of the matrix.

In the determination of the HCPC sand content, it is imperative to ensure that the flow rate remains within an appropriate range while also contemplating its impact on the compressive strength subsequent to carbonation curing. Synthesizing the aforementioned analyses, an aggregate volume fraction of 40% has been identified as the optimal process condition, striking a balance between workability and mechanical performance.

### 3.5. Chloride Permeation Resistance

Resistance to chloride attack is an important characterization method for testing the durability of materials [34]. The chloride penetration region of concrete products is determined by color development of AgNO_3_ solution, with chloride-penetrated regions appearing white and unpenetrated regions appearing yellow.

**Figure 8:**
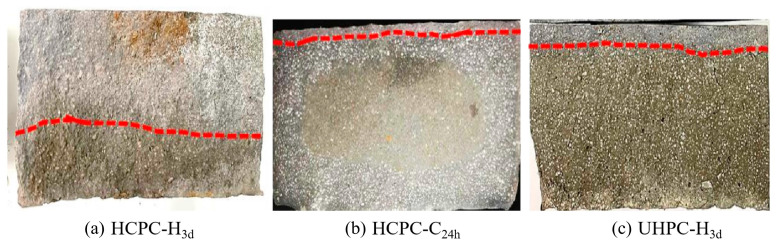
The specimens showed color after RCM testing under different curing conditions.

As demonstrated in Figure 8, which displays the color development images from the Rapid Chloride Migration (RCM) test under various carbonation curing conditions, where the red line indicates the demarcation line between developed and non-developed colours. It is noteworthy that a distinct non-carbonated area is present in the center of the section post-curing. The color produced by AgNO_3_ solution spray tanning resembles that of the carbonated region, and upon closer inspection, the boundary of the chloride infiltration zone can still be discerned, where HCPC has a shallower depth of chloride erosion after 24 h of carbonation curing after pretreatment that UHPC after 3 d of hydration maintenance displays. Table 3 presents the calculation of the chloride ion diffusion coefficient, revealing that carbonation curing significantly enhances the resistance to chloride ion penetration in the products. This is due to the carbonation byproducts that fill most pores and decrease the overall matrix porosity. Notably, a dense outer layer effectively blocks the pathways for chloride ions to penetrate the concrete, endowing the post-carbonation cured products with superior impermeability to chloride ions [35].

### 3.6. Freeze–Thaw Cycle Test

#### 3.6.1. Appearance Damage Analysis

**Figure 9:**
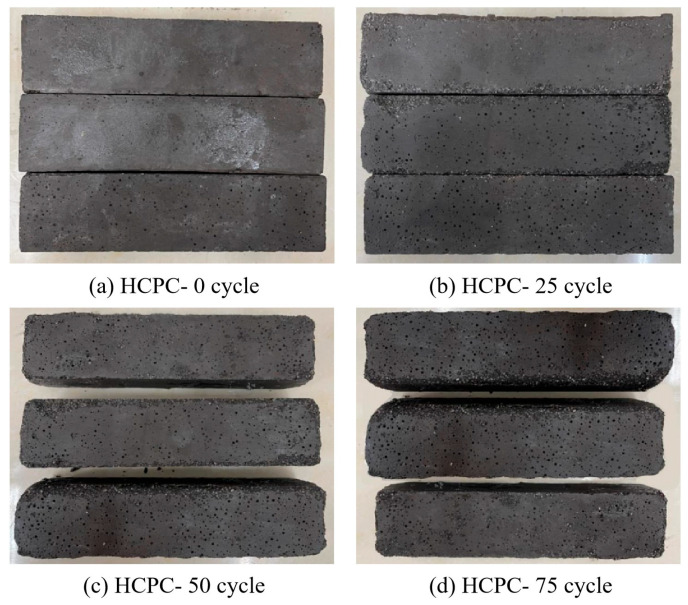
Appearance of HCPC at different numbers of freeze–thaw cycles.

Figure 9 illustrates the alterations in the physical appearance of HCPC specimens subjected to multiple freeze–thaw cycles. The visual evidence indicates that the degree of spalling on the HCPC specimens escalates with an increasing number of freeze–thaw cycles. After enduring 25 cycles, the specimens exhibited minor spalling on the ribs, with no substantial damage attributable to the freeze–thaw process. However, upon completion of 50 cycles, the specimens’ edges began to manifest severe damage, characterized by pronounced flaking that exposed the underlying quartz sand aggregate. Post 75 cycles, there was a marked reduction in the specimens’ dimensions; increased surface residue was shed, and the corners had entirely disappeared. The depiction of the specimens after 100 cycles is omitted, but it is noted that at this juncture, the rate of mass loss surpassed acceptable thresholds, the internal structure was severely compromised, and the surface layer of some specimens had been completely eroded.

Despite these observations, when juxtaposed with conventional cement mortars under standard curing conditions, HCPC still demonstrates a heightened resistance to frost action [36]. This comparative resilience underscores the enhanced durability of HCPC in harsh environmental conditions, which is a critical consideration in the evaluation of its performance.

#### 3.6.2. Quality Loss Rate

**Figure 10:**
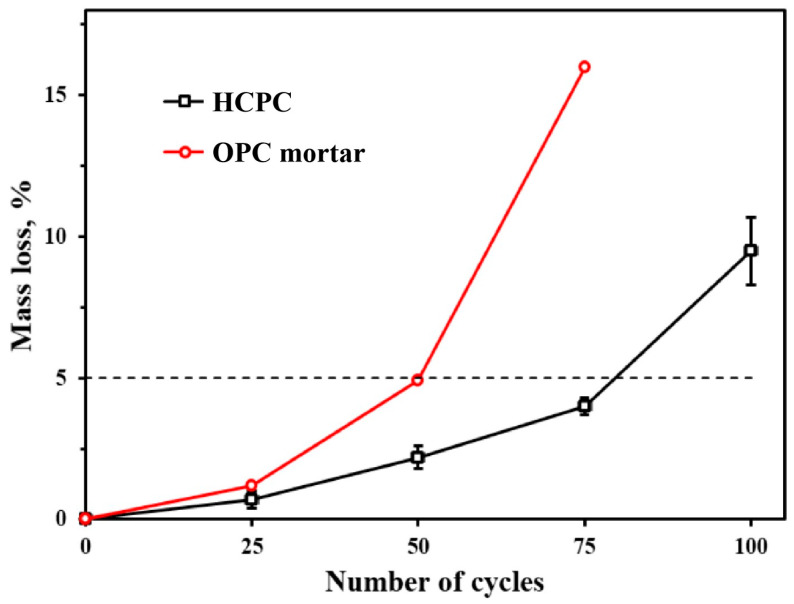
Mass loss rates of HCPC and OPC mortars with different numbers of freeze–thaw cycles.

Figure 10 shows the mass loss rates of both HCPC and ordinary Portland cement mortar (OPC) specimens following various freeze–thaw cycles. The mass loss rates for OPC mortar specimens, as depicted in the figure, were reported by other researchers [36]. The comparative analysis clearly demonstrates that HCPC exhibits enhanced frost resistance relative to OPC mortar. Even after enduring 75 freeze–thaw cycles, HCPC maintains compliance with the maximum permissible mass loss threshold for the freeze–thaw cycle test, a benchmark that OPC mortar surpasses after only 50 cycles. As the cycle count escalates, the specimens incur more pronounced damage, culminating in a weakened internal structure and an accelerated degradation rate due to surface spalling.

Upon reaching 100 freeze–thaw cycles, the mass loss rate of HCPC has also exceeded the 5% threshold, peaking at 9.5%. The data’s increased margin of error at this point further underscores that the specimens have sustained considerable damage, with substantial surface layer peeling amplifying the variability in the results.

#### 3.6.3. Compressive Strength Loss Rate

**Figure 11:**
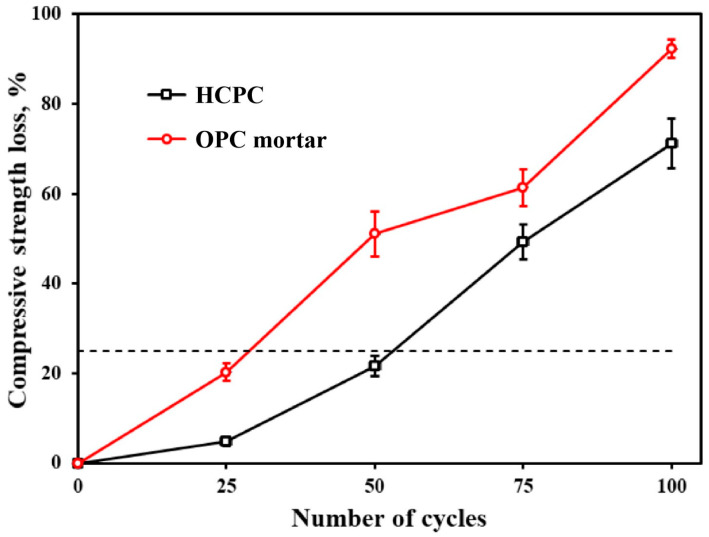
Loss of compressive strength of HCPC and OPC mortars at different numbers of freeze–thaw cycles.

Figure 11 illustrates the relationship between the compressive strength loss rate of HCPC and OPC mortar specimens in relation to the number of freeze–thaw cycles they undergo. The data indicate that HCPC reaches a critical threshold in compressive strength loss much earlier than it does for the maximum permissible mass loss rate, achieving this critical point after only 50 cycles. The ongoing damage to the internal structure of the HCPC specimens, exacerbated by the freeze–thaw process and the expansion due to pore solution icing, leads to an enlargement of the pore volume. This results in the gradual peeling off of the outer surface layer and corners of the HCPC specimens. Such peeling significantly impacts the compressive strength testing, causing a notable reduction in the measured compressive strength values.

Despite these effects, it is clear that the compressive strength loss rate of HCPC is consistently lower than that observed in OPC mortar specimens. This disparity underscores the superior frost resistance of HCPC, suggesting that it can better withstand the deleterious effects of freeze–thaw cycles compared to conventional OPC mortar.

### 3.7. High-Temperature Resistance

For traditional cementitious and concrete materials, a degradation in mechanical properties is commonly observed under conditions of elevated temperature [37,38]. This deterioration is predominantly due to the thermal decomposition of hydration products. Notably, the decomposition temperatures for C-S-H and calcium hydroxide, integral components of concrete, are approximately 300 °C and 400 °C, respectively. Studies have demonstrated that the implementation of carbonation curing can significantly bolster the high-temperature performance of concrete products [39]. The carbonation curing process results in the formation of carbonation products, including calcium carbonate and silica gel. These products exhibit enhanced thermal stability compared to the hydration products of cement, such as the C-S-H gel and calcium hydroxide. The superior thermal stability of calcium carbonate and silica gel is attributed to their chemical structures, which are more resistant to thermal breakdown. Consequently, the introduction of these carbonation products through curing can improve the concrete’s resistance to high-temperature-induced degradation, thereby maintaining its structural integrity and mechanical properties under such conditions.

**Figure 12:**
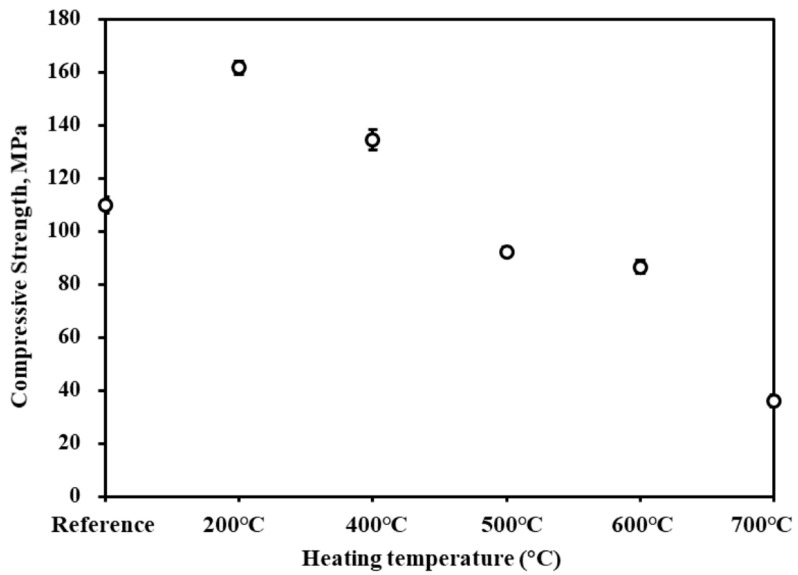
Compressive strength of HCPC after different temperature treatments.

Figure 12 shows the changes in compressive strength of HCPC after heat treatment at different temperatures, from which it can be seen that the compressive strength of HCPC is greatly improved when the temperature is increased to 200 °C–400 °C, and the strength at 200 °C is 160 MPa, which is 45% higher than the compressive strength of untreated HCPC. One of the reasons for this may be that under high temperature conditions, the unhydrated particles in the specimen will accelerate hydration, thus further increasing the compressive strength. When the temperature rises to 500 °C, the unreacted Ca(OH)_2_ inside the HCPC decomposes, resulting in internal microcracks and a slight reduction in strength. At 600 °C, a small amount of amorphous calcium carbonate inside the HCPC undergoes decomposition reaction and the compressive strength is further reduced. When the temperature reaches 700 °C, the compressive strength of HCPC decreases rapidly, and the strength is only 40% of that of the control specimen. At this time, a large amount of calcium carbonate decomposes inside the HCPC, a large number of cracks appear, and the damage of HCPC is obvious; another reason for the decrease in the compressive strength at this temperature is the decomposition of amorphous silica gel. The sequence of chemical reactions and physical alterations that occur within the concrete matrix as the temperature increases is complex and multifaceted. The initial increase in strength due to accelerated hydration is eventually overshadowed by the detrimental effects of decomposition, which include the formation of microcracks and the loss of structural integrity due to the breakdown of key binding agents.

### 3.8. Further Discussion

This subsection provides a comprehensive discussion and comparison of the findings from existing studies, with a primary focus on the analysis of preparation methods, water–binder ratios, sample sizes, curing conditions, and compressive strength. The literature has broadly classified the carbonation curing of steel slag into two principal methodologies based on the molding technique: compression molding and pouring molding. In compression molding of steel slag, the necessity for powder fluidity is obviated, enabling the direct compaction into a dense billet under meticulously controlled conditions. However, this method is restricted to uniform dimensions and is associated with higher costs due to the stringent process requirements. On the other hand, pouring molding, which employs higher water-to-solid ratios to ensure fluidity, may be impeded by excess moisture during the CO_2_ curing phase, potentially leading to a reduction in strength. Nonetheless, pouring molding offers distinct advantages, such as the flexibility to produce various sizes and shapes and a more straightforward preparation process for carbonized steel slag products.

Table 4 presents a comparative analysis of performance statistics, highlighting that the low water–binder ratio casting and molding method utilized in this study amalgamates the benefits of compressive strength and durability properties that surpass the levels reported in existing research. Additionally, this method confers the capability to fabricate products conforming to the shape of the mold, offering a valuable reference for the industrial application of this technology.

## 4. Conclusions

The primary objective of this study is to harness steel slag as a valuable resource in the production of HCPC, suitable for use in prefabricated construction components. To achieve this, an optimal mix ratio was meticulously formulated to develop a preparation method for HCPC. The HCPC was fabricated using a pouring and molding technique that significantly incorporated steel slag as an admixture. Subsequent to the fabrication process, the compressive strength and durability performance of the HCPC, following carbonation curing, were systematically assessed. This evaluation aimed to provide a theoretical framework and practical technical guidance for the application of HCPC in engineering contexts. The following conclusions were derived from the study’s findings:

(1) Reduction in the w/b ratio substantially augmented the compressive strength of HCPC following carbonation curing, achieving a peak of 104.9 MPa at a w/b ratio of 0.18. Whereas, an increase in SF dosage and the corresponding decrease in cement content lead to the decrease in strength before carbonation curing, and the increase in SF dosage can improve the compactness of the HCPC system, so that the compressive strength after carbonation curing can be improved.

(2) Permeation resistance of HCPC was evaluated using the anti-chloride ion permeability coefficient. The chloride ion diffusion coefficient for HCPC was measured at 0.78 × 10^−12^ m^2^/s, which represents a nearly 95% reduction compared to the uncarbonated group, indicating a significant improvement in permeability resistance.

(3) Frost resistance of HCPC was investigated and characterized by parameters such as mass loss rate and compressive strength loss rate. After 12 h of carbonation curing, the frost resistance of HCPC was superior to that of OPC mortar specimens. Following 100 cycles of freezing and thawing, all indices of the HCPC specimens were found to be below the threshold values set by standards, and no cracking damage was observed.

(4) HCPC demonstrated resistance to high temperatures, with its compressive strength notably increasing within the temperature range of 200 °C to 400 °C. 

## Figures and Tables

**Table 1:** Chemical composition of cement, CSS, and SF (%).

Chemical Composition	CaO	SiO_2_	Al_2_O_3_	Fe_2_O_3_	SO_3_	MgO	K_2_O	Na_2_O	LOI
Cement	65.21	21.27	4.57	3.25	2.48	1.62	0.76	0.08	0.76
CSS	46.84	12.38	5.72	25.56	0.25	5.61	0.81	0.13	2.70
SF	0.39	94.57	0.51	0.23	0.26	0.65	1.05	0.34	2.00

Note: CSS means converted steel slag, SF means silica fume, LOI means loss on ignition.

**Table 2:** Mixing ratio design.

No.	Binder/wt.%			w/b	Sand (Volume Fraction/%)	SP kg/m^3^
	Cement	SF	CSS			
1	6	4	90	0.4	40	0
2				0.3		4.7
3				0.25		9.06
4				0.18		44.9
5	20	0	80	0.18	40	45.5
6	17	3				45.2
7	14	6				44.9
8	12	8				44.7
9	12	8	80	0.18	40	44.7
10	6	4	90			44.9
11	3	2	95			45.3
12	0	0	100			45.5
13	6	4	90	0.18	0	75.9
14					20	60.4
15					30	52.7
16					40	44.9

Note: Amount of SP is the quality added to adjust the fluidity of mortar. W/b means water-to-binder ratio, CSS means converted steel slag, SF means silica fume, SP means a polycarboxylic acid-based high-efficiency reducer.

**Table 3:** Rapid chloride diffusion coefficient of concrete under different curing conditions.

	U (V)	x_d_ (mm)	D_nssm_ (×10^−12^ m^2^/s)
HCPC-H_3d_	25	31	17.5(±1.6)
HCPC-C_24h_	25	2.1	0.78(±0.15)
UHPC-H_3d_	25	3.5	1.3(±0.18)

Note: D_nssm_ means the fast chloride migration coefficient of the concrete specimen (×10^−12^ m^2^/s), U means the applied voltage (V), x_d_ means the average depth of penetration of the tested chloride ions (mm). -H_3d_ means 3 d of hydration curing only, −C_24h_ means carbonation curing for 24 h after pre-treatment as taken in the previous section.

**Table 4:** Statistics on the results of carbonization studies of steel slag products.

Raw Materials	Preparation Method	Water–Binder Ratio	Product Size	Curing Condition	Compressive Strength/MPa
Steel slag Gypsum [8]	compression molding	0.2	Φ20 × 10 mm	CO_2_ 20 ± 3%, 1 d	32
KOBM slag [21]	compression molding	0.15	Φ15 × 30 mm	CO_2_ 99.5%, 0.15 MPa, 2 h	80.5
KOBM slag [22]	compression molding	0.1	Φ15 × 20 mm	CO_2_ 99.5%, 0.15 MPa, 2 h	46
Steel slag [23]	compression molding	0.1	61 × 61 × 33 mm	CO_2_ 17%, 24 h, 0.15 MPa, 20 °C	40
BOF slag EAF slag [24]	compression molding	0.1	Φ23 × 20 mm	CO_2_ 100%, 1 MPa, 50 °C, 4 h	47.2
AOD slag [40]	compression molding	0.15	40 × 40 × 40 mm	CO_2_ 100%, 15 h, 0.8 MPa, 80 °C	59.8
BOFS(80%) P.II 52.5 [16]	pouring molding	0.54	100 × 100 × 100 mm	CO_2_ 99.9%, 0.1 MPa, 1 d	33.5
Steel slag Slaked lime River sand [25]	pouring molding	0.5	40 × 40 × 160 mm	CO_2_ 20%, 20 ± 5 °C	22.7
AODS OPC [26]	pouring molding	0.5	-	CO_2_ 5%, 20 °C, 7 d	24.9
BOFS(30%) P.I 42.5 Steel slag [28]	pouring molding	0.5	40 × 40 × 160 mm	CO_2_ 99.9%, 90 °C, 7 h	24.5
BOFS(80%) P.II 52.5 Steel slag [41]	pouring molding	0.4	20 × 20 × 20 mm	CO_2_ 99.9%, 0.1 MPa, 1 d	59.5
This paper Steel slag	pouring molding	0.18	40 × 40 × 40 mm	CO_2_ 99.9%, 0.4 MPa, 12 h	104.9

Note: KOBM means Klockner Oxygen Blown Maxhutte steel slag, BOF means Basic Oxygen Furnace steel slag, EAF means Electric Arc Furnace steel slag, AOD means Argon Oxygen Decarburization steel slag.

## Data Availability

Data is contained within the article.
